# Supplementary material for: Vasorelaxant Activity of Salvia hispanica L.: Involvement of the Nitric Oxide Pathway in Its Pharmacological Mechanism
Source: Molecules. 2023 Aug 24;28(17):6225. doi: 10.3390/molecules28176225 (PMC10488739; doi:10.3390/molecules28176225)
Supplement: Supplementary file 1 [file molecules-28-06225-s001.zip › molecules-2501082-supplementary.pdf]

Supplementary Materials

Luis A. Herbert-Doctor [1,†], Amanda Sánchez-Recillas [1,†], Rolffy Ortiz-Andrade [1,*], Emanuel Hernández-Núñez [2], Jesús Alfredo Araujo-León [3], Tania Isolina Coral-Martínez [4], Nubia Noemi Cob-Calan [5], Maira Rubi Segura Campos [6] and Samuel Estrada-Soto [7]

1 Laboratorio de Farmacología, Facultad de Química, Universidad Autónoma de Yucatán, Mérida 97069, Yucatan, Mexico; luis.herbert@correo.uady.mx (L.A.H.-D.); amanda.sanchezrecillas@gmail.com (A.S.-R.)
2 Departamento de Recursos del Mar, Centro de Investigación y de Estudios Avanzados del IPN, Mérida 97310, Yucatán, Mexico; emanuel.hernandez@cinvestav.mx
3 Unidad de Bioquímica y Biología Molecular de Plantas, Centro de Investigación Cinetífica de Yucatán, A.C., Mérida 97205, Yucatan, Mexico; jalfredoaraujo@gmail.com
4 Laboratorio de Cromatografía, Facultad de Química, Universidad Autónoma de Yucatán, Mérida 97069, Yucatan, Mexico; tcoral@correo.uady.mx
5 Instituto Tecnológico Superior de Calkiní en el Estado de Campeche, Calkiní 24900, Campeche, Mexico; nubia.cob@itconkal.edu.mx
6 Facultad de Ingeniería Química, Universidad Autónoma de Yucatán, Mérida 97203, Yucatán, Mexico; maira.segura@correo.uady.mx
7 Facultad de Farmacia, Universidad Autónoma del Estado de Morelos, Cuernavaca 62209, Morelos, Mexico; enoch@uaem.mx
* Correspondence: rolffy@correo.uady.mx; Tel./Fax: +52-999-9225711 or +52-999-9225716
† These authors contributed equally to this work.

Table S1. Experimental and reported (in the same deuterated solvent; $CDCl_3$ for linoleic acid, and hexyl octanoate and CD_3OD for mannitol) ^{13}C-NMR chemical shift values of natural products predicted in the dereplication analyses.

Position	Linoleic acid		Mannitol	
	**Experimental**	**Reported** [1]	**Experimenta l**	**Reported** [2]
1	179.15	178.90	64.60	65.00
2	33.90	33.80	72.49	72.60
3	24.64	24.60	71.17	71.00
4	29.06	29.00	71.05	71.00
5	29.14	29.10	73.04	72.60
6	29.32	29.10	64.41	65.00
7	29.56	29.70		
8	27.16	27.20		
9	130.05	130.00		
10	128.05	128.00		
11	25.59	25.60		
12	127.75	127.90		
13	130.33	130.00		
14	27.40	27.20		
15	29.67	29.30		
16	31.50	31.50		
17	22.65	22.60		
18	14.17	14.10		

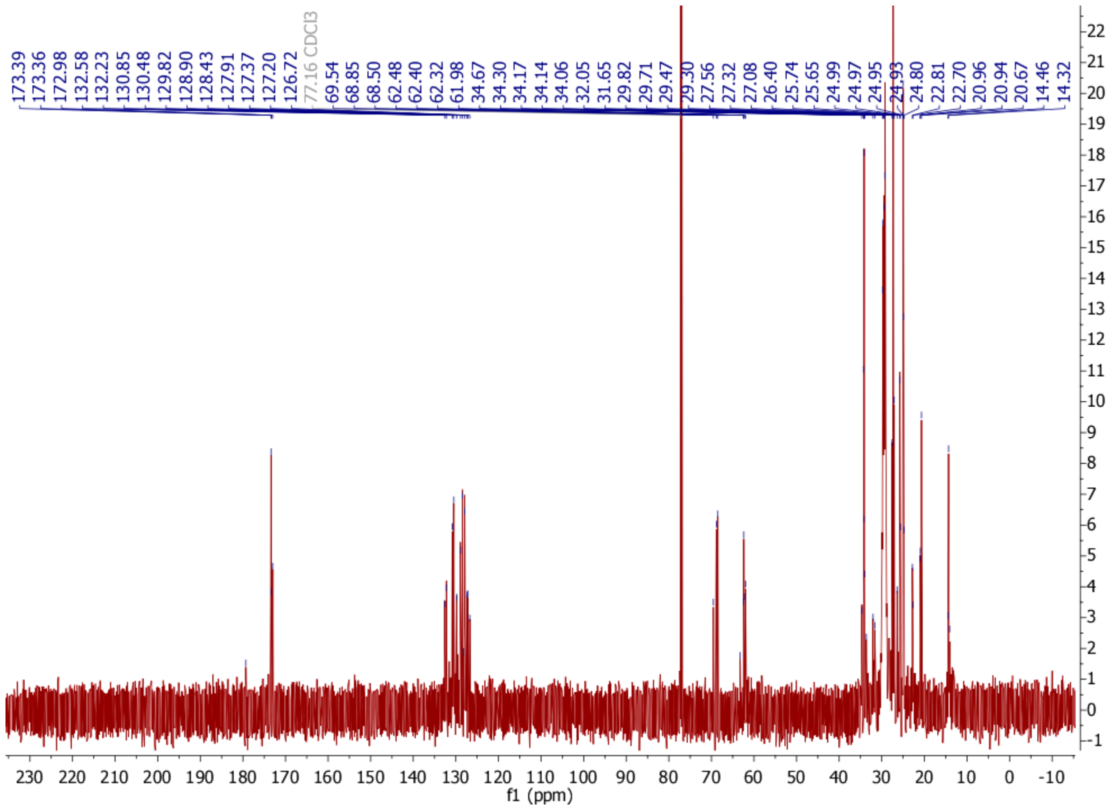

Figure S1. ^{13}C-NMR (150 MHz, $CDCl_3$) spectrum of hexanic extract (**HE*Sh***) from *S. hispanica* seeds.

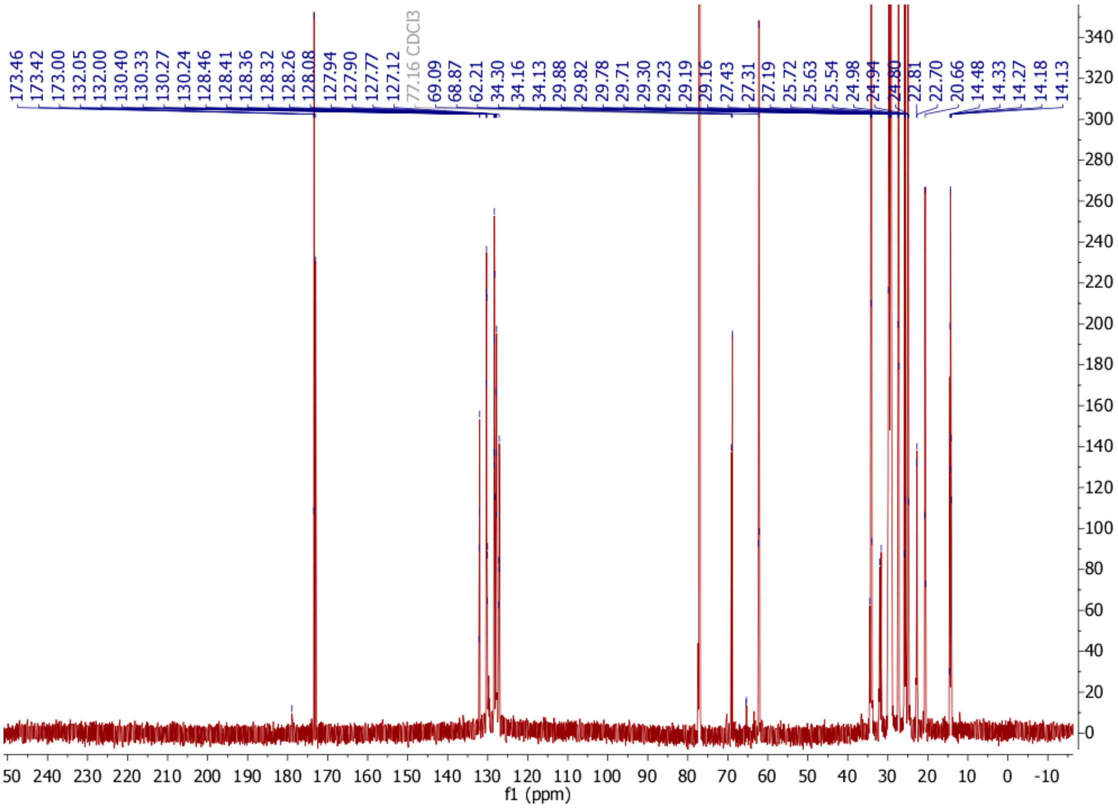

Figure S2. ^{13}C-NMR (150 MHz, $CDCl_3$) spectrum of chloroformic extract (**DE*Sh***) from *S. hispanica* seeds.

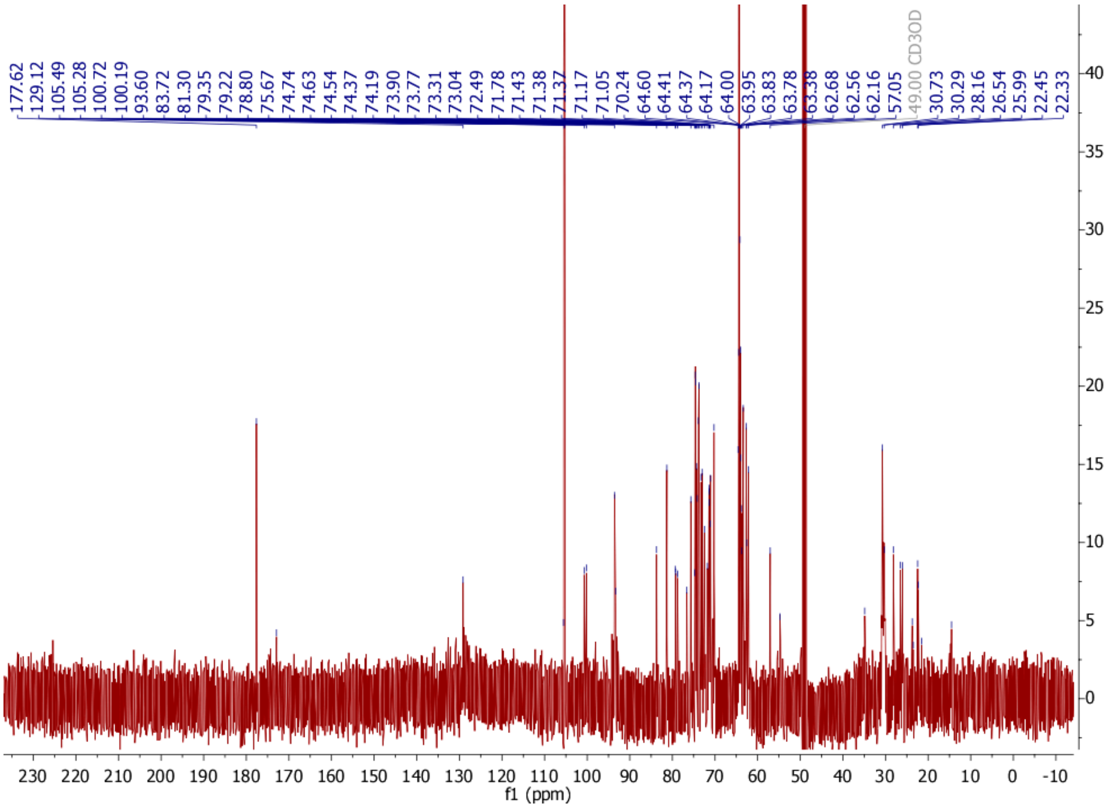

Figure S3. ^{13}C-NMR (150 MHz, CD_3OD) spectrum of methanolic extract (**ME*Sh***) from *S. hispanica* seeds.

References

1. Marwah, R.G.; Fatope, M.O.; Deadman, M.L.; Al-Maqbali, Y.M.; Husband, J. Musanahol: A New Aureonitol-Related Metabolite from a *Chaetomium* Sp. *Tetrahedron* **2007**, *63*, 8174–8180, doi:10.1016/J.TET.2007.05.119.
2. Kim, J.S.; Jin, C.K.; Sang, H.S.; Eun, J.L.; Jin, W.Y.; Bae, K.H.; Kun, H.S.; Hyun, P.K.; Sam, S.K.; Hyeun, W.C. Chemical Constituents of the Root of *Dystaenia Takeshimana* and Their Anti-Inflammatory Activity. *Arch Pharm Res* **2006**, *29*, 617–623, doi:10.1007/BF02968244/METRICS
